# Feasibility, acceptability, and effectiveness of a package of interventions to improve the performance of health workers in the Democratic Republic of Congo to deliver adolescent and youth sexual and reproductive health services

**DOI:** 10.1080/16549716.2025.2540687

**Published:** 2025-08-26

**Authors:** Eric Mafuta, Kasra Zarei, Landry Egbende, Benito Kazenza, Sheri Bastien, Venkatraman Chandra-Mouli

**Affiliations:** a Department of Health Systems Policy and Management, University of Kinshasa School of Public Health, Kinshasa, Democratic Republic of the Congo; b Department of Global Public Health, Karolinska Institutet, Stockholm, Sweden; c Department of Nutrition, University of Kinshasa School of Public Health, Kinshasa, Democratic Republic of the Congo; d Department of Public Health Science, Norwegian University of Life Sciences, Ås, Norway; e Cumming School of Medicine, Department of Community Health Sciences, University of Calgary, Calgary, Canada; f Department of Sexual and Reproductive Health and Research, World Health Organization, Geneva, Switzerland; g Independent Learner, Communicator, Advisor, Teacher, and Supporter of Adolescent Advocacy, Research, and Action, Geneva, Switzerland

**Keywords:** Adolescent sexual and reproductive health, workforce performance, implementation science, sub-Saharan Africa, mixed methods

## Abstract

**Background:**

Adolescents and youth (AY) in the Democratic Republic of Congo (DRC) face significant sexual and reproductive health (SRH) challenges and require access to quality health services.

**Objective:**

This study assesses the feasibility, acceptability, and effectiveness of a package of interventions to improve the performance of health workers (HWs) providing SRH services to AY in the DRC.

**Methods:**

This mixed-methods process and outcome evaluation was conducted between January and July 2021 in 30 health facilities in Mbuji-Mayi and Kinshasa. Data were collected through interviews with 25 health facility managers, surveys of 54 HWs, focus groups with 29 HWs, and interviews with 16 AY mystery clients and 18 female AY (10–24 years) health facility service users. Qualitative and quantitative data were analyzed using thematic content analysis and descriptive statistics respectively.

**Results:**

Health facility managers reported that the package of interventions was feasible to implement, with concerns about resource shortcomings which were outside the scope of the implementation. From both quantitative and qualitative findings, health facility managers and HWs perceived the package of interventions as acceptable and effective in improving HW competencies, building positive attitudes towards providing SRH services to AY, building HW motivation, and fostering an enabling work environment as illustrated by improved communication, mentorship and constructive feedback between managers and HWs, and motivation, confidence and collaboration among HWs. AY clients reported overall satisfaction with the services they received, but some reported experiencing inappropriate comments and judgmental attitudes.

**Conclusions:**

The package of interventions was perceived as feasible and acceptable by health facility managers and HWs, and effective in improving HW knowledge, skills, attitudes and motivation, and ultimately in providing SRH services to AY. Future efforts are needed to validate the findings in different contexts and with other professionals, e.g. teachers, and to strengthen and amplify the package of interventions.

## Paper Context

- **Main findings:** The findings indicate that the health facility managers, health workers and AY reported that the package of interventions was feasible to implement, with concerns about shortcomings in terms of resources which were not intended to be included in the implementation, as well as acceptable and effective in improving health worker performance to provide quality SRH services to AY.
- **Added knowledge:** This study found that it is still possible to improve health worker performance to provide quality SRH services without providing financial incentives, but with a comprehensive package of interventions including job descriptions, training and refresher training sessions, desk reference tools, supportive supervision and collaborative learning sessions.
- **Global health impact for policy and action**: We expect that the insights from our study on enhancing the competencies and attitudes of health workers, as well as fostering a supportive physical and social environment, could provide valuable guidance for health programs aimed at improving the knowledge, attitudes, skills, competencies, motivation and performance of health workers to provide quality SRH services for AY.

## Background

A trained, competent, and empathetic workforce is critical to providing high-quality adolescent-friendly promotive, preventive, and curative health services [1]. This is true for health service provision broadly [2], and is especially true in relation to providing sexual and reproductive health (SRH) services, including contraceptive services [3]. In this context, there is a pressing need to improve both the clinical and interpersonal performance of health workers (HWs). Previous research has shown that factors such as inadequate professional competency, judgmental attitudes and behaviors, and low professional motivation can lead to poor clinical and interpersonal performance, which can hinder the provision to and utilization of SRH services by adolescents and young people (AY) [4]. Fundamentally, individual factors (such as knowledge and understanding and attitudes), factors in the immediate environment (such as the availability of medicines, commodities and equipment) and those in the wider environment (such as the institutional and social context) together affect HW motivation and performance [5] and must be addressed [2]. Addressing this web of factors that operate at multiple levels has been shown to improve the quality of health service provision and increase utilization of SRH services by AY, which further improves short- and long-term SRH outcomes [6].

Nevertheless, the evidence base for HW behavior change interventions and related factors that influence HW behavior is still limited [3]. Although understandings of which interventions targeting HW motivation and performance are effective in improving AY SRH and workforce performance has steadily grown [4], studies that have contributed to this understanding have primarily been small-scale, with limited sample sizes and primarily addressed factors only at the individual and workplace environment levels that influence HW behavior. Gaps remain in terms of the perceived feasibility, acceptability, and effectiveness of such interventions in resource-constrained settings and on how to deliver them with equity and quality at scale in such settings. Often only standalone interventions including single session didactic training have been implemented in large-scale programs [7], without employing other proven and promising interventions to address the larger array of factors described above. These interventions include non-financial incentives including collaborative learning and teamwork [8], recognition [8] and human resource management tools including supportive supervision [2,9]. Approaches that combine a package of evidence-based intervention strategies have been shown to be more effective than piecemeal approaches in improving workforce motivation and performance [10,11]. Expert recommendations to advance programming of HW behavior change related to family planning and SRH in AY emphasize the need to address the diversity of HWs and their environments employing approaches that go beyond training, while also expanding their focus to include both HW and client-side interventions, support rather than blame HWs, and more rigorously measure HW behavior change [3]. Principles of person-centered care must also be incorporated into health systems, to ensure that they are responsive to the diverse needs of AY [12].

The objective of this mixed-methods study was to determine the feasibility, acceptability and effectiveness of a package of interventions implemented in two cities and in six health zones in the DRC [13], aimed at improving the competencies of HWs by enhancing their knowledge and skills on how to deliver health interventions, and understanding of when to use them; building positive attitudes in providing SRH services to AY in general and specific interventions; and motivating HWs to do the best they can through deploying their competencies and using the resources at their disposal. The specific study objectives and research/evaluation questions have been described previously [13] and focus on perceptions of the feasibility and acceptability of the package of interventions (in-service training and retraining, desk reference tools, i.e. job aids, supportive supervision, collaborative learning and joint problem identification and problem-solving) by health facility managers and HWs, perceptions of acceptability of AY clients receiving services from HWs trained through the package of interventions, and the perceptions of all stakeholders in the effectiveness of the package of interventions in improving HWs’ competencies and attitudes related to responding to the SRH needs of AY clients.

## Methods

### Study design

The study adopted a mixed methods process and outcome evaluation design to assess the feasibility, acceptability, and effectiveness of a package of interventions to improve HW performance in delivering SRH services to AY [13]. The package of interventions was implemented from 2017 to 2020. The collaborative learning and supportive supervision sessions that took place were implemented with a frequency of once a month. The refresher training occurred once in a block session (2 days at each site). While the study intended to adopt a time series design, due to the end of funding from the Global Fund, this plan could not be carried forward. Data presented for this study were collected at one time point. The implementation research study design has been previously described, and primarily targeted HWs, health facility managers and beneficiaries of project activities, namely AY [13]. The study focused on exploring the perceptions of health facility managers who took part in project activities, HWs trained and supported as part of the package of interventions, and the AY clients in relation to the services received.

### Setting

Data collection was conducted in three health zones in Kinshasa and three health zones in Mbuji-Mayi in Kasaï Oriental, which were selected by the implementing partners of the Global Fund, namely the National Program against AIDS (PNLS), Cordaid, National Adolescent Health Program (PNSA) in the DRC and Réseau National des ONG pour le Développement de la Femme (RENADEF), based on the baseline of HIV prevalence rates among women aged 15–24, and the presence of health services already supported by the GFATM through existing grants.

### Study population and participants

The study involved HWs, health facility managers and non-minor AY clients at the health facilities. The participants were selected according to a purposive sampling strategy. Participants in the health zones studied were selected with a sampling strategy as previously described [13], ensuring adequate coverage of rural and urban facilities. The interviews and focus group discussions consisted of a convenience sample of HWs and facility managers who had received training (Supplemental Material).

### Qualitative and quantitative data collection

Qualitative data were collected using several data collection methods including 25 individual semi-structured interviews with health facility managers, 4 focus group discussions with HWs, 18 exit interviews with AY clients, and 16 interviews with AY mystery clients (Supplemental Table 1) [13]. For quantitative data collection, a survey was administered one time between April and May 2021 to a sample of 54 HWs at health facilities participating in the intervention between January and July 2021. As noted above, additional rounds of data collection were planned, but could not be carried out because of funding constraints.

The data collection tools consisted of interview and focus group discussion guides, a survey for HWs, an interview guide with mystery clients, and an exit interview guide for use with AY clients [13]. The data collection tools were validated (Supplemental Methodology) and translated from English to French, and back-translated to verify the translation. Data was collected first in Kinshasa and then in Mbuji-Mayi, by the trained local research teams in each respective location. Interviews with mystery clients and exit interviews with AY clients were conducted last. Mystery clients were young women aged 18–20, recruited from the Congolese Youth Association Network (RACOJ) and who received a 3-day training in the method.

### Qualitative data analysis

Interviews and focus group discussions were audio recorded, transcribed into French and analyzed using thematic analysis [14]. Thematic coding was conducted independently by four team members to ensure inter-rater reliability. Discrepancies were resolved through discussion and consensus. The interviews that were conducted in local languages, particularly Lingala in Kinshasa and Tshiluba in Mbuji-Mayi, especially with mystery clients and during exit interviews, were also recorded and transcribed into French, and subsequently all transcripts were translated into English. These transcripts were verified by the research assistants by listening to the recordings and comparing them with the transcripts.

### Quantitative data analysis

Survey data were uploaded from the tablets to a secure cloud server using the SurveyCTO application and then exported to Stata (version 14) for analysis. Descriptive statistical analyses consisted of summarizing the data for categorical variables by study site. The proportions of HWs from participating health facilities in Kinshasa were compared to those from Mbuji-Mayi using Pearson’s chi-square test. When the chi-square test was not applicable, Fisher’s exact test or the chi-square test of likelihood were used to compare proportions. While the two study sites did not vary substantially in terms of the intervention implemented, these statistical tests were conducted to assess any quantitative differences in survey responses between the two study sites.

### Triangulation of methods and results

To strengthen the validity of the findings and enhance the depth of the analysis, the study used a mixed methods approach which included qualitative and quantitative data in order to reduce the limitations of relying on one type of evidence alone, and to enable triangulation of the main study findings [13]. Qualitative insights were used to contextualize and help explain quantitative results, providing a more comprehensive understanding of the interventions’ impact. For instance, qualitative themes related to HW motivation and attitudes towards AY SRH, and perceptions of components of the package of interventions were compared with survey findings. Mystery client interviews allowed for triangulation with adolescent client exit interviews to provide a robust assessment of service delivery and AY client perceptions and experiences. As mystery clients and AY exit interview clients were the recipients of services whose quality was influenced in part by the package of interventions, they were only asked questions related to the acceptability and effectiveness of care and services received, and not the feasibility of the package of interventions as in the case of health facility managers and HWs.

### Ethical considerations

The research protocol was approved by the Ethics Committee of the Kinshasa School of Public Health (N°ESP/CE/149/2020; N°ESP/CE/149B/2022) and the WHO-ERC (ERC.0003228). Participation in the study was subject to informed consent and minors were not included in the study. Each participant received an explanation of the study, its objectives and expected results. They were informed that participation was voluntary. Focus group participants received reimbursement for their travel and the mystery clients who were part of the research team received a stipend. The data were processed and analyzed in such a way as to remove all direct and indirect information that could identify the respondents [13]. Participation in the study posed minimal risk to respondents and respondents had no direct benefits from participating in this study. Potential mystery clients were initially screened to identify those who have previously experienced GBV-related trauma. If they reported having experienced GBV, they were referred for counselling and excluded from the selection process to prevent psychosocial harm as a result of participation in the study. Mystery clients who were selected received a three-day training provided by the University of Kinshasa, which was based on standardized, best-practice approaches to training mystery clients with modules which focused on ethics and confidentiality, what to expect in terms of standard of care, how to engage in role play, how to ask for information and advice concerning SRH issues, how to conduct observations and memory techniques, and how to complete the checklists and reporting requirements. To safeguard their confidentiality, mystery clients were given a pseudonym and fictional birth date, and they carried a letter of participation in case they faced any questions about their role in the study. Finally, to ensure support and assistance for the mystery clients was available if needed, a trained staff member from the University of Kinshasa was present on site at the health facilities, to step in if needed.

## Results

An overview of the socio-demographic characteristics of the study participants is provided in Supplemental Table 2. The findings presented are structured according to the order of the study objectives and research questions. First, we present findings from the qualitative data related to perceptions and experiences of feasibility, acceptability, and effectiveness of the package of interventions, followed by the results of the quantitative survey of HWs.

### Qualitative findings

The findings from the interviews with health facility managers, focus group discussions with HWs, and interviews with mystery clients and AY exit interview clients are presented in relation to the feasibility, acceptability, and effectiveness of the package of interventions, by each stakeholder. Thematic saturation was reached with additional data collection not yielding any new themes.

#### Feasibility

##### Perceptions and experiences of health facility managers

Health facility managers reported that activities implemented as part of the package of interventions did not impose an additional burden on their work schedule. They reported that their learnings from the training programs contributed to better self-organization by the HWs and better organization of workflow within the health facility. However, multiple health facility managers affirmed that the human and financial resources allocated for the implementation of the package of interventions were insufficient and should be increased to improve the feasibility of implementing the package of interventions. They also emphasized that the implementation activities should be extended to other HWs in the same and other health facilities, including other provinces so that more HWs and ultimately AY could benefit. Some health facilities did not benefit from all the resources they wanted to receive (teaching materials, etc.) and had not yet implemented the full package of interventions due to lack of funding and essential resources.

We can expand everywhere because if the intervention is extended everywhere, it will give good returns… (Male, 43 years old)

##### Perceptions and experiences of health workers

In focus group discussions, most HWs reported that from their perspective, the package of interventions was feasible. However, some HWs noted shortcomings in the implementation echoing perspectives shared by the health facility managers: primarily related to a lack of resources and time. HWs reported that a lack of resources including human resources (i.e. trained HWs), medical supplies and workspaces reserved for adolescent SRH services created challenges that affected the feasibility of the package of interventions. One HW also reported a lack of relevant flowcharts, checklists, and frameworks to address the specific SRH needs of AY. As previously mentioned, it is possible that the scope of the contents of the package of interventions (which did not focus on improving amenities, providing or repairing equipment, or providing medicines and supplies^12^) was perhaps not clear to HWs, thus creating misunderstanding and a mismatch in expectations.

A challenge I would say is regarding human resources, the lack or deficit of the number of trained people … if someone leaves, the others will have difficulty doing it. As far as materials are concerned, we have no inputs, no drugs. Financially, nothing was done until the end of the project. (Male, 30 years old)

Additionally, some HWs noting the positive potential of the package of interventions, and particularly the supportive supervision and collaborative learning components, expressed a desire for the geographic scope of the project to be expanded so that other health facilities and HWs in other parts of the DRC could benefit.

#### Acceptability

##### Perceptions and experiences of health facility managers

The package of interventions was considered acceptable overall by most of the health facility managers, mostly because it was perceived as being relevant and helped establish a framework for better management of AY SRH problems. Furthermore, interviewees reported that the package of interventions – particularly components such as the training, supportive supervision, and collaborative learning – also helped to positively impact the knowledge, skills, attitudes, competencies, and motivation of HWs related to the planning, management, and provision of services and care to AY.

The whole package was useful because it was new material. We didn’t have all that information. Everything was really helpful. (Male, 40 years old)

##### Perceptions and experiences of health workers

Through focus group discussions, the HWs unanimously reported that they highly appreciated the package of interventions because it met a real need for both HWs and the community.

[The package of interventions was] Very acceptable for the community because it allowed us to significantly reduce the cases of STIs in the community. (Male, 50 years old)

However, some HWs considered that the training time provided as part of the package of interventions was insufficient to adequately cover all contents to be covered, and they subsequently did not have enough time to assimilate the learning. Still, most HWs reported in focus group discussions that they appreciated how the training material was adapted to their needs to help improve their work.

… the training helped us dot the ‘i‘s’, the youth represent the future of the country. (Male, 35 years old)

Several HWs affirmed that they found the information and tools provided as part of the package of interventions to help with providing care and services tailored to the needs of adolescents.

Yes, yes, at my place the guides are really useful because in front of a young person I draw a lesson or a theme from the technical sheet and we discuss [it] with him. (Male, 45 years old)

##### Perceptions and experiences of AY exit clients

Exit interview clients reported overall positive perceptions of the acceptability of care received by AY clients. Most exit interview clients generally reported having been welcomed and well received at health facilities, having their rights respected, not feeling judged based on age or appearance, and receiving SRH services and care that meets their needs. For example, an exit interview client reported:

The nurse welcomed me very well in relation to what I had come for, because she was attentive to what I was telling her; when I arrived, I found her, she was working, she gave up what she was doing, she came to answer me, then she gave me a place to sit and we started talking, I told her my problem and she also told me what I could do. (Female, 21 years old)

Overall, AY clients reported that the HWs showed empathy, welcoming attitudes, encouragement and that they were taken to a setting where they could receive information and services in a confidential manner. Some exit interview clients specifically reported that confidentiality wasn’t always discussed by HWs. Still, most clients similarly reported that they were welcomed and received the requested information services from respectful HWs. For instance, an exit interview client reported:

… I even liked them because they gave me the advice that I hadn’t been given when I came before. I don’t know if because I was in pain I couldn’t listen to other advice, but today I really wanted this advice. I was treated, I was given advice and that’s it. (Female, 19 years old)

##### Perceptions and experiences of mystery clients

Consistent with the perceptions of the exit interview clients, overall, most mystery clients (14 out of 16 mystery clients, 5/6 in Kinshasa and 9/10 in Mbuji-Mayi) similarly reported that they were well received by the HWs who showed empathy, welcoming attitudes, encouragement and that they were taken to a setting where they could receive information and services in a confidential manner.

Yes, I was comfortable, and she listened to me attentively, she let me talk until the end. (Female, 18 years old)

However, a minority of clients reported not feeling welcomed or receiving encouraging attitudes, experiencing a delay in their appointment, perceiving HWs to be overwhelmed upon arrival, not receiving satisfactory services from the point of reception to interacting with HWs, or receiving comments that they felt were inappropriate. For example, a mystery client reported:

She didn’t have an encouraging attitude, the way she spoke to me, sometimes she looked at me and she sulked at me, I thought to myself how can I have the courage to speak with a person who looks at me and sulks at me first of all. The sulking there discouraged me a little. (Female, 18 years old)

#### Effectiveness

##### Perceptions and experiences of health facility managers

Health facility managers interviewed reported that the package of interventions was effective in leading to several positive changes in their workplace – including AY-friendly services, enhanced collaboration, improved job descriptions and workplace organization.

After the training we received, there was a very big change in our health center. First, there was an addition of adolescent-friendly services. Among the tasks we were working on, we added a service adapted to teenagers. Previously there were only services for women and children. Afterwards there was a modification at the level of the reception. Previously we welcomed adolescents in a different way but since the training we have changed. Before when an adolescent comes for the consultation, I make them wait like all the other patients. But since the training, I can’t make the adolescent wait long at the center. If they come, it’s for a few minutes, and then we don’t talk together in front of everyone. There is a setting reserved for the reception of adolescents, it is in this setting that I have to go and talk with them. (Female, 50 years old)

Furthermore, health facility managers emphasized the importance of job aids such as guidelines in improving the knowledge and skills of their HWs who provide SRH care and services to AY and building empathic and nonjudgemental attitudes so that the services they provide meet the needs and rights of AY.

This training gave us certain guidelines: how can we approach a young person, how can we guide the teenager, and how can we respond to or solve a problem that young people bring to us. (Male, 40 years old)

##### Perceptions and experiences of health workers

Through focus group discussions, HWs reported that due to the training they received as part of the package of interventions, which emphasized the importance of providing care and services that meet the needs and preferences of AY, they changed the way they offer AY care and services.

Training was of paramount importance, before we received young people like everyone else, but with training, we learned not to hide anything from young people, we learned to structure the care of young people, we learned how to teach young people to use condoms, we learned who a young person is. (Female, 37 years old)

The majority of HWs reported that the introduction of supportive supervision improved communication and interaction within the health facility between health facility managers and HWs and further solidified the understanding of certain aspects not assimilated during the training.

At my place, for example, there was at the beginning a supervisor who came to guide me on what I didn’t understand … and that motivated me in the way of working and communicating with him. (Female, 34 years old)

Additionally, HWs reported that the collaborative learning approach was applied in most settings and enabled them to improve their skills through discussions and sharing experiences with their colleagues, which further contributed to facilitating positive interactions between adolescents and the HWs. Additionally, the collaborative learning sessions helped improve other aspects such as confidentiality.

Collaborative learning has helped us improve our experience or change experiences with others at these learning meetings. We meet together, everyone has their own community problems or their health issues, but we exchange experiences, we can take the case or even one day I can find myself in front of the case that my colleague or colleague had presented during the learning meetings and we capture this experience to practice with others to deepen our knowledge. (Female, 30 years old)

##### Perceptions and experiences of AY exit interview clients

Regarding the effectiveness of care received by AY clients, AY exit interview clients reported that the HWs asked each of them for the purpose of their visit and provided advice. The majority of clients (15 out of 16 mystery clients, 6/6 in Kinshasa and 9/10 in Mbuji-Mayi) reported that their needs were met at either at the health facility or through a referral. Furthermore, the majority of AY clients said that they could understand information provided by the HWs, and that the HWs took the time to check that they understood all the information provided to them. For example, one AY exit interview client reported:

Yes, he always asked me whether I understood what he was saying in relation to the consultation and the advice he was giving me. (Female, 21 years old)

Nearly all clients reported that the consultation successfully was conducted by a HW that displayed professionalism and who was able to establish rapport. For example, one client reported:

… Basically what I appreciated was more the availability of the doctors and their kindness. The person who received me was very professional, courteous and willing to listen. So that gave me confidence and I even took the time to tell him some things that were a bit personal. I really liked her. (Female, 15 years old)

##### Perceptions and experiences of mystery clients

Mystery clients overall reported similar perceptions concerning the effectiveness of care received to AY exit interview clients. A minority of them reported that HWs did not ask them if they had a personal concern they wanted to discuss. Mystery clients generally reported that they received the service they came for at the health facility or through a referral, for instance, the use of condoms to avoid STIs and unwanted pregnancies, and the use of contraceptive pills when indicated. Some mystery clients said they did not know how to properly use condoms, and consequently, some HWs demonstrated to clients how to use condoms correctly to avoid STIs and unwanted pregnancies. For example, a mystery client reported:

Yeah he told me if it [intercourse] happens again, I have to talk to my boyfriend that we don’t have to do it – or have to do it only with the condom to protect us, so protect me and protect us, protect us all. He advised me, but he didn’t support it, he said you have to be very careful. (Female, 19 years old)

While this quote illustrates that the personal values of HWs may have influenced the services provided, there is no indication that any personal values in this case or other situations in the implementation of the package of interventions prevented the ARSH services from being delivered to the client (e.g. counseling about condom and oral contraceptive use). Nearly all clients reported that that the HWs informed them not to hesitate to come back in case of problems and were thus happy to respond to their subsequent concerns. For example, one mystery client reported:

Yes, he told me, if your friend or you need more information, you and your friend, come back here, I’m here for you. (Female, 18 years old)

### Quantitative findings

The HW survey was conducted among 24 HWs in Kinshasa and 30 HWs in Mbuji-Mayi both from health facilities that participated in the project, with overall comparable sociodemographic characteristics at both study sites, as indicated in Table 1. The age of the majority of HWs was between 25 and 44 years old (Table 1) and just over half were women (63.0%). Among the HWs surveyed, almost all in Kinshasa (95.8%) and all in Mbuji-Mayi received training on how to provide ARSH care, with varying proportions reporting benefitting from the components of the package of interventions which was intended to include the provision of in-service training, job aids, coordination strengthening, supportive supervision and collaborative learning sessions (Table 1).

**Table 1.** Characteristics of the health workers who participated in the study.

	Kinshasa		Mbuji-Mayi		All		
	(*n* = 24)	%	(*n* = 30)	%	(*n* = 54)	%	*p*
Age							0.258
Under 25 years	8	33.3	3	10.0	11	20.4	
25–34 years	5	20.8	9	30.0	14	25.9	
35–44 years	6	25.0	8	26.7	14	25.9	
45–54 years	4	16.7	6	20.0	10	18.5	
55 years and more	1	4.2	4	13.3	5	9.3	
Gender							0.614
Male	8	33.3	12	40.0	20	37.0	
Female	16	66.7	18	60.0	34	63.0	
Have received training on how to provide specific care to adolescents, including young women							0.259
No	1	4.2	0	0.0	1	1.9	
Yes	23	95.8	30	100.0	53	98.2	
Watched a video							0.091
No	13	54.2	10	33.3	23	42.6	
Yes	10	41.7	20	66.7	30	55.6	
Missing	1	4.2	0	0	1	1.9	
Have attended a lecture or conference							<0.001
No	17	70.8	8	26.7	25	46.3	
Yes	6	25.0	22	73.3	28	51.9	
Missing	1	4.2	0	0	1	1.9	
Have attended a skills-based training or workshop							0.683
No	4	16.7	4	13.3	8	14.8	
Yes	19	79.2	26	86.7	45	83.3	
Missing	1	4.2	0	0	1	1.9	
Have attended collaborative learning sessions							<0.001
No	9	37.5	1	3.3	10	18.5	
Yes	14	58.3	29	96.7	43	79.6	
Missing	1	4.2	0	0	1	1.9	
Have had other training							0.758
No	14	58.3	17	56.7	31	57.4	
Yes	9	37.5	13	43.3	22	40.7	
Missing	1	4.2	0	0	1	1.9	
Have changes made to job descriptions related to the provision of adolescent care in your health facility within the last 6 months							0.003
No	17	70.8	10	33.3	27	50.0	
Yes	6	25.0	20	66.7	26	48.1	
Missing	1	4.2	0	0	1	1.9	
Have had new job aids such as checklists, guidelines or other decision support tools introduced to your health facility related to the provision of adolescent care in the last 6 months							0.152
No	16	66.7	15	50.0	31	57.4	
Yes	7	29.2	15	50.0	22	40.7	
Missing	1	4.2	0	0	1	1.9	
Have received supportive supervision in the past 6 months that differed in content and tone from what you previously received from your health facility manager							0.414
No	12	50.0	19	63.3	31	57.4	
Yes	11	45.8	11	36.7	22	40.7	
Missing	1	4.2	0	0	1	1.9	

Of the HWs surveyed in both Kinshasa and Mbuji-Mayi, the majority considered multiple factors important for their motivation including seeing the job as extremely important to patients (94.4%) and more than a job (94.4%), the sense of duty of the HWs to take care of their patients (94.4%), and the fact that being a HW was an integral part of their personality (87.0%). Generally, characteristics didn’t vary between both study sites (Table 2). Regarding enabling factors in the work environment, the majority of HWs surveyed reported factors including feeling appreciated when patients are grateful (100%), getting along well with their supervisors at work (100%), and that the feedback they get from their manager helps them improve their work (96.3%). The proportion of the responses of the HWs to the different survey items about work environment factors were generally comparable between Kinshasa and Mbuji-Mayi (Table 3).

**Table 2.** General information on the activities and motivations of health workers.

	Kinshasa (*n* = 24)		Mbuji-Mayi (*n* = 30)		All (*n* = 54)		
	n	%	n	%	n	%	*p*
**Because the work that I do is very interesting**							0.177
Not important	0	0.0	4	13.3	4	7.4	
Neutral	3	12.5	3	10.0	6	11.1	
Important	21	87.5	23	76.7	44	81.5	
**Because I like the challenges I face in my work**							0.188
Not important	0	0.0	3	10.0	3	5.6	
Neutral	9	37.5	7	23.3	16	29.6	
Important	15	62.5	20	66.7	35	64.8	
**Because I enjoy interacting with many people every day**							0.452
Not important	4	16.7	9	30.0	13	24.1	
Neutral	3	12.5	2	6.7	5	9.3	
Important	17	70.8	19	63.3	36	66.7	
**Because being a health worker is a fundamental part of who I am**							0.968
Not important	2	8.3	3	10.0	5	9.3	
Neutral	1	4.2	1	3.3	2	3.7	
Important	21	87.5	26	86.7	47	87.0	
**Because my work is more than a job, it’s a mission**							0.659
Not important	1	4.2	1	3.3	2	3.7	
Neutral	0	0.0	1	3.3	1	1.9	
Important	23	95.8	28	93.3	51	94.4	
**Because my work is extremely important for my patients**							0.690
Not important	1	4.2	2	6.7	3	5.6	
Neutral	0	0.0	0	0.0	0	0.0	
Important	23	95.8	28	93.3	51	94.4	
**Because I want to make a difference in people’s lives** Not important	1	4.2	11	36.7	12	22.2	0.016
Neutral	5	20.8	5	16.7	10	18.5	
Important	18	75.0	14	46.7	32	59.3	
**Because this job fits my personal values very well**							0.082
Not important	1	4.2	7	23.3	8	14.8	
Neutral	1	4.2	3	10.0	4	7.4	
Important	22	91.7	20	66.7	42	77.8	
**Because my reputation depends on my work**							0.188
Not important	3	12.5	10	33.3	13	24.1	
Neutral	3	12.5	2	6.7	5	9.3	
Important	18	75.0	18	60.0	36	66.7	
**Because my work makes me feel proud of myself**							0.336
Not important	1	4.2	4	13.3	5	9.3	
Neutral	1	4.2	3	10.0	4	7.4	
Important	22	91.7	23	76.7	45	83.3	
**Because it is my duty to care for my patients**							0.690
Not important	0	0.0	0	0.0	0	0.0	
Neutral	1	4.2	2	6.7	3	5.6	
Important	23	95.8	28	93.3	51	94.4	
**Because of the appreciation I receive from my patients and the community**							0.130
Not important	2	8.3	8	26.7	10	18.5	
Neutral	3	12.5	1	3.3	4	7.4	
Important	19	79.2	21	70.0	40	74.1	
**So I don’t let my team down**							0.025
Not important	1	4.2	10	33.3	11	20.4	
Neutral	2	8.3	3	10.0	5	9.3	
Important	21	87.5	17	56.7	38	70.4	
**Because my supervisor recognizes and appreciates me**							0.004
Not important	3	12.5	17	56.7	20	37.0	
Neutral	4	16.7	2	6.7	6	11.1	
Important	17	70.8	11	36.7	28	51.9	
Not important	6	25.0	15	50.0	21	38.9	
Neutral	9	37.5	8	26.7	17	31.5	
Important	9	37.5	7	23.3	16	29.6	
**In order to be able to provide for my family**							0.827
Not important	7	29.2	11	36.7	18	33.3	
Neutral	5	20.8	5	16.7	10	18.5	
Important	12	50.0	14	46.7	26	48.2	
**Because of the financial security my job provides me with**							0.680
Not important	13	54.2	13	43.3	26	48.2	
Neutral	6	25.0	8	26.7	14	25.9	
Important	5	20.8	9	30.0	14	25.9

**Table 3.** Information on the work environment of health workers.

	Kinshasa (*n* = 24)		Mbuji-Mayi (*n* = 30)		All (*n* = 54)		
	n	%	n	%	n	%	*p*
**My job duties and responsibilities are clear and precise**							0.032
Disagree	2	8.3	0	0.0	2	3.7	
Neutral	3	12.5	0	0.0	3	5.6	
Agree	19	79.2	30	100.0	49	90.7	
**Relevant guidelines are readily available at this facility**							0.025
Disagree	5	20.8	3	10.0	8	14.8	
Neutral	4	16.7	0	0.0	4	7.4	
Agree	15	62.5	27	90.0	42	77.8	
**I often feel alone when I have to make difficult decisions about services**							0.536
Disagree	18	75.0	21	70.0	39	72.2	
Neutral	2	8.3	1	3.3	3	5.6	
Agree	4	16.7	8	26.7	12	22.2	
**I have regular access to relevant training to maintain my skills**							0.025
Disagree	5	20.8	3	10.0	8	14.8	
Neutral	4	16.7	0	0.0	4	7.4	
Agree	15	62.5	27	90.0	42	77.8	
**My performance is evaluated regularly**							0.407
Disagree	4	16.7	8	26.7	12	22.2	
Neutral	4	16.7	2	6.7	6	11.1	
Agree	16	66.7	20	66.7	36	66.7	
**Promotions don’t depend on how well or badly one performs at work**							0.177
Disagree	10	41.7	20	66.7	30	55.6	
Neutral	5	20.8	3	10.0	8	14.8	
Agree	9	37.5	7	23.3	16	29.6	
**It’s hard for me to talk openly to my superiors about how things are really going at work** Disagree	16	66.7	28	93.3	44	81.5	0.039
Neutral	1	4.2	0	0.0	1	1.9	
Agree	7	29.2	2	6.7	9	16.7	
**Suggestions made by workers on how to improve**							0.006
Disagree	10	41.7	25	83.3	35	64.8	
Neutral	6	25.0	2	6.7	8	14.8	
Agree	8	33.3	3	10.0	11	20.4	
**The management of the facilities does not concern me much**							0.495
Disagree	14	58.3	22	73.3	36	66.7	
Neutral	3	12.5	2	6.7	5	9.3	
Agree	7	29.2	6	20.0	13	24.1	
**Our rights as health workers are generally not respected**							0.730
Disagree	7	29.2	11	36.7	18	33.3	
Neutral	4	16.7	6	20.0	10	18.5	
Agree	13	54.2	13	43.3	26	48.2	
**I don’t get feedback from my superiors, so it’s hard to improve**							< 0.001
Disagree	14	58.3	30	100.0	44	81.5	
Neutral	4	16.7	0	0.0	4	7.4	
Agree	6	25.0	0	0.0	6	11.1	
**The feedback I get from my manager helps me improve my work**							0.107
Disagree	0	0.0	0	0.0	0	0.0	
Neutral	2	8.3	0	0.0	2	3.7	
Agree	22	91.7	30	100.0	52	96.3	
**Good performance is recognized by our superiors**							0.133
Disagree	1	4.2	1	3.3	2	3.7	
Neutral	3	12.5	0	0.0	3	5.6	
Agree	20	83.3	29	96.7	49	90.7	
**This facility has a Fair system for rewarding staff**							0.229
Disagree	15	62.5	13	43.3	28	51.9	
Neutral	2	8.3	2	6.7	4	7.4	
Agree	7	29.2	15	50.0	22	40.7	
**Some Team members work well, but others don’t**							0.338
Disagree	16	66.7	17	56.7	33	61.1	
Neutral	6	25.0	6	20.0	12	22.2	
Agree	2	8.3	7	23.3	9	16.7	
**Our facility has clear goals that we are working on**							0.519
Disagree	1	4.2	0	0.0	1	1.9	
Neutral	1	4.2	1	3.3	2	3.7	
Agree	22	91.7	29	96.7	51	94.4	
**I want to use new tools to improve my performance**							
Disagree	0	0.0	0	0.0	0	0.0	
Neutral	0	0.0	0	0.0	0	0.0	
Agree	24	100.0	30	100.0	54	100.0	
**This facility enjoys a good reputation in the community**							0.197
Disagree	0	0.0	0	0.0	0	0.0	
Neutral	0	0.0	2	6.7	2	3.7	
Agree	24	100.0	28	93.3	52	96.3	
**I understand how my work contributes to the general objectives of the Establishment**							
Disagree	0	0.0	0	0.0	0	0.0	
Neutral	0	0.0	0	0.0	0	0.0	
Agree	24	100.0	30	100.0	54	100.0	
**I feel appreciated when patients are grateful**							0.367
Disagree	0	0.0	0	0.0	0	0.0	
Neutral	0	0.0	1	3.3	1	1.9	
Agree	24	100.0	29	96.7	53	98.2	
**I am proud to work for this health facility**							0.158
Disagree	2	8.3	1	3.3	3	5.6	
Neutral	4	16.7	1	3.3	5	9.3	
Agree	18	75.0	28	93.3	46	85.2	
**I intend to leave this Institution as soon as I can find another job**							0.158
Disagree	5	20.8	10	33.3	15	27.8	
Neutral	6	25.0	9	30.0	15	27.8	
Agree	13	54.2	11	36.7	24	44.4	
**Overall, I am very satisfied with my work in this Establishment**							0.339
Disagree	2	8.3	7	23.3	9	16.7	
Neutral	1	4.2	1	3.3	2	3.7	
Agree	21	87.5	22	73.3	43	79.6	
**I try to get along well with other health care staff because**							0.259
Disagree	0	0.0	0	0.0	0	0.0	
Neutral	1	4.2	0	0.0	1	1.9	
Agree	23	95.8	30	100.0	53	98.2	
**I get along well with my superiors at work**							0.259
Disagree	0	0.0	0	0.0	0	0.0	
Neutral	1	4.2	0	0.0	1	1.9	
Agree	23	95.8	30	100.0	53	98.2	

Regarding attitudes towards adolescents, more than half of the HWs reported that a pregnant girl should be allowed to continue schooling (92.6%), adolescents should be given contraceptive information and counseling before they become sexually active (90.7%), if a schoolgirl is sexually active she should be allowed to use contraceptives (85.2%), and adolescents have the same rights to family planning information and services as any other older or married clients (83.3%). Proportions of the responses of the HWs to the different survey items about attitudes towards adolescents were generally comparable between Kinshasa and Mbuji-Mayi (Table 4). Regarding attitudes towards sexual violence and intimate partner violence, 96.3% of HWs reported that how they respond to an adolescent/young woman who has suffered violence from a partner or sexual abuse is very important (Table 5). There were few observed differences at both study settings in HW responses to questions concerning attitudes towards sexual violence and intimate partner violence (Table 5).

**Table 4.** Information on attitudes towards adolescents and youth.

	Kinshasa (*n* = 24)		Mbuji-Mayi (*n* = 30)		All (*n* = 54)		
	n	%	n	%	n	%	*p*
**I would first recommend unmarried adolescent girls to abstain from sex when they ask for contraceptives**							0.285
Disagree	7	29.2	15	50.0	22	40.7	
Neutral	3	12.5	2	6.7	5	9.3	
Agree	14	58.3	13	43.3	27	50.0	
**If a schoolgirl is sexually active she should be allowed to use contraceptives**							0.720
Disagree	2	8.3	3	10.0	5	9.3	
Neutral	2	8.3	1	3.3	3	5.6	
Agree	20	83.3	26	86.7	46	85.2	
**Adolescents should be given contraceptive information and counseling before they become sexually active**							0.914
Disagree	1	4.2	2	6.7	3	5.6	
Neutral	1	4.2	1	3.3	2	3.7	
Agree	22	91.7	27	90.0	49	90.7	
**Providing contraceptives to unmarried adolescents promotes sexual promiscuity**							0.581
Disagree	12	50.0	13	43.3	25	46.3	
Neutral	3	12.5	2	6.7	5	9.3	
Agree	9	37.5	15	50.0	24	44.4	
**My personal beliefs influence my ability to provide sexual and reproductive health information and services to adolescents**							0.645
Disagree	11	45.8	17	56.7	28	51.9	
Neutral	2	8.3	3	10.0	5	9.3	
Agree	11	45.8	10	33.3	21	38.9	
**Adolescents have the same rights to family planning information and services as any other older or married clients**							0.079
Disagree	1	4.2	5	16.7	6	11.1	
Neutral	0	0.00	3	10.0	3	5.6	
Agree	23	95.8	22	73.3	45	83.3	
**A pregnant girl should be allowed to continue schooling** Disagree	1	4.2	1	3.3	2	3.7	0.266
Neutral	2	8.3	0	0.0	2	3.7	
Agree	21	87.5	29	96.7	50	92.6	
**An adolescent girl with a genital ulcer is likely to be promiscuous**							0.011
Disagree	8	3.3	18	60.0	26	48.2	
Neutral	11	45.8	3	10.0	14	25.9	
Agree	5	20.8	9	30.0	14	25.9

**Table 5.** Attitudes towards sexual violence and intimate partner violence (IPV).

	Kinshasa (*n* = 24)		Mbuji-Mayi (*n* = 30)		All (*n* = 54)		
	n	%	n	%	n	%	*p*
**As a health worker, how I respond to an adolescent/young woman who has suffered violence from a partner or sexual abuse is very important**							0.273
Disagree	1	4.2	0	0.0	1	1.9	
Neutral	1	4.2	0	0.0	1	1.9	
Agree	22	91.7	30	100.0	52	96.3	
**An adolescent/young woman subjected to violence will deny that she has been abused if I ask her about it**							0.198
Disagree	7	29.2	14	46.7	21	38.9	
Neutral	7	29.2	10	33.3	17	31.5	
Agree	10	41.7	6	20.0	16	29.6	
**Intimate partner violence is a private matter and outsiders should not interfere**							0.720
Disagree	20	83.3	26	86.7	46	85.2	
Neutral	1	4.2	2	6.7	3	5.6	
Agree	3	12.5	2	6.7	5	9.3	
**Sometimes, being abused, assaulted or raped is the woman’s own fault**							0.056
Disagree	15	62.5	26	86.7	41	75.9	
Neutral	6	25.0	4	13.3	10	18.5	
Agree	3	12.5	0	0.0	3	5.6	
**If the woman had defended herself, she could have avoided being raped**							0.351
Disagree	10	41.7	16	53.3	26	48.2	
Neutral	4	16.7	7	23.3	11	20.4	
Agree	10	41.7	7	23.3	17	31.5	
**I should convince an adolescent/young woman subjected to intimate partner violence to leave her violent relationship**							0.012
Disagree	11	45.8	24	80.0	35	64.8	
Neutral	4	16.7	0	0.0	4	7.4	
Agree	9	37.5	6	20.0	15	27.8	
**If a woman does not leave her violent partner, she deserves to be abused** Disagree	18	75.0	24	80.0	42	77.8	0.908
Neutral	1	4.2	1	3.3	2	3.7	
Agree	5	20.8	5	16.7	10	18.5	
**I would feel uncomfortable asking an adolescent/young woman about violence**							0.137
Disagree	21	87.5	30	100.0	51	94.4	
Neutral	1	4.2	0	0.0	1	1.9	
Agree	2	8.3	0	0.0	2	3.7	

Regarding perceptions of the effectiveness of components of the package of interventions, more than half of the HWs reported positive perceptions including that their ability to perform their job and provide quality care to adolescents has improved since the introduction of the collaborative learning model (92.6%), that the model helped to ensure a system of support among HWs (88.9%), and that HWs at their facility solved problems as a group rather than alone now as a result of the collaborative learning model (88.9%). Overall, the majority of survey respondents affirmed that the supportive supervision and collaborative learning model has helped foster communication, a more collaborative learning environment, mentorship, and HW motivation. Once again, there were few observed differences at both study settings in HWs’ responses to questions concerning the perceptions of the other components of the interventions used in this study (Table 6). Even survey items with significantly different proportions between both study sites showed the same trend among the majority at both study settings (Table 6).

**Table 6.** Perceptions of the other components of the package of interventions (supportive supervision and collaborative learning model).

	Kinshasa (*n* = 24)		Mbuji-Mayi (*n* = 30)		All (*n* = 54)		
	n	%	n	%	n	%	*p*
**Communication with my manager has improved since the introduction of supportive supervision**							0.158
Disagree	2	8.3	1	3.3	3	5.6	
Neutral	4	16.7	1	3.3	5	9.3	
Agree	18	75.0	28	93.3	46	85.2	
**There is greater emphasis on mentorship and facilitation since the introduction of supportive supervision**							0.191
Disagree	3	12.5	1	3.3	4	7.4	
Neutral	4	16.7	2	6.7	6	11.1	
Agree	17	70.8	27	90.0	44	81.5	
**I receive more constructive feedback from my manager since the introduction of supportive supervision**							0.223
Disagree	5	20.8	3	10.0	8	14.8	
Neutral	5	20.8	3	10.0	8	14.8	
Agree	14	54.2	24	80.0	38	70.4	
**My motivation to perform my job has increased since the introduction of supportive supervision**							0.024
Disagree	6	25.0	3	10.0	9	16.7	
Neutral	5	20.8	1	3.3	6	11.1	
Agree	13	54.2	26	86.7	39	72.2	
**There is a more collaborative environment at this health facility since we started using the collaborative learning model**							0.012
Disagree	5	20.8	1	3.3	6	11.1	
Neutral	3	12.5	0	0.0	3	5.6	
Agree	16	66.7	29	96.7	45	83.3	
**Health workers at this facility solve problems as a group rather than alone now as a result of the collaborative learning model**							0.132
Disagree	3	12.5	0	0.0	3	5.6	
Neutral	1	4.2	2	6.7	3	5.6	
Agree	20	83.3	28	93.3	48	88.9	
**My confidence in my ability to perform my job and provide quality care to adolescents has improved since the introduction of the collaborative learning model** Disagree	2	8.3	0	0.0	2	3.7	0.266
Neutral	1	4.2	1	3.3	2	3.7	
Agree	21	87.5	29	96.7	50	92.6	
**The collaborative learning model has helped to ensure a system of support among health workers**							0.259
Disagree	2	8.3	0	0.0	2	3.7	
Neutral	2	8.3	2	6.7	4	7.4	
Agree	20	83.3	28	93.3	48	88.9	

## Discussion

The study evaluated the feasibility, acceptability and effectiveness of a package of interventions – consisting of proven approaches – aimed at improving the performance of HWs, which is a composite of their competencies and attitudes in providing SRH services to AY, and their motivation to do their best to ensure the provision of quality SRH services to AY in health facilities of the DRC in the cities of Kinshasa and Mbuji-Mayi. The main results of this study were that the combination of approaches used in the package of interventions – the provision of in-service training, job aids, and collaborative learning sessions as well as supportive supervision and strengthening coordination – was overall feasible to implement in the health facilities, despite some concerns about human and financial resources, and with high levels of acceptability with respect to contributing to improving individual competencies, attitudes and establishing an enabling work environment and thereby the provision of quality SRH services to AY.

A package of interventions – including collaborative learning – targeting healthcare providers to improve the quality of AY SRH services, has been shown to be feasible, acceptable and effective in other contexts as well. For example, Lesco et al. found that collaborative learning was both doable and worth doing in the context of a national initiative to scale up youth-friendly health centres [15]. The collaborative learning approach is particularly effective because it allows providers to identify and discuss challenges experienced in providing care to adolescents, and to share their experiences in managing these challenges [15], which could in turn, help improve performance on specific health indicators. In DRC as in Moldova, collaborative learning has been implemented at low-cost and enables learning that is aligned with local realities. For the package of interventions implemented in this study, the collaborative learning sessions continued beyond the duration of the project, speaking to the sustainability of this component, although this aspect was not directly assessed in this study. Additionally, the nature of the organization of the health system in DRC includes regular meetings to validate data collected from different health zones. These routine activities bring together healthcare providers and serve as opportunities for experience-sharing and exchanges [16,17]. This could facilitate the adoption of the collaborative learning approach, which builds on existing practices, although here the focus is specifically on AY SRH.

While the intention of the package of interventions was never to provide inputs such as medication, equipment, and supplies, this was perhaps not clearly communicated to all stakeholders which could have led to a misunderstanding and a mismatch in expectations. For instance, several health facility managers and HWs were anticipating that the project would build specific spaces for AY, provide the health facilities with medications and clinical and laboratory equipment and materials, and grant a financial subsidy to cover training activities for HWs. Demotivation and a lack of medicines, supplies, and equipment have long been barriers to the provision of services in the DRC as in other countries [18–20]. In this context, service providers expect either financial or material support in order to carry out their role. It is essential that future program implementation and research should ensure clear communication with all stakeholders as to what support will and will not be provided to ensure better alignment of expectations through clearer communication and stakeholder engagement from the outset.

Additionally, the package of interventions was perceived to be effective in terms of improving the competencies of HWs by improving their knowledge, skills, and understanding of when to use an intervention; building positive attitudes in providing SRH services to AY; and building HW motivation to do the best they can, by deploying the competencies they have, and the tools they have at their disposal. The overall positive perceptions of the package of interventions were also reflected by the AY who participated in the mystery client and exit interviews and generally reported having been well-received and well-treated during their visits to health facilities. Despite this, some AY clients reported a few inappropriate statements or practices from HWs suggesting the persistence of certain social and gender norms and indicating there is room for improvement regarding addressing judgmental attitudes among HWs. Social norms generally take time to change and require carefully tailored and targeted interventions delivered over time. This requires sustained support from decision makers at different levels [20]. To assist AY in navigating their sexual and reproductive lives, program designers and implementers must consider the role of underlying societal norms in a more strategic and context-specific manner [20,21].

The mixed-methods study design allowed for triangulation of the main study findings – primarily through incorporating a variety of interviews, focus groups, and surveys, and mitigating the limitations of relying on one type of evidence alone [13]. In general, the study showed that the package of interventions was feasible and acceptable for HWs and managers of health facilities, evidenced by the interviews, FGDs and the proportion of surveyed HWs that reported successful implementation of the package of interventions (modified job descriptions, trainings, provision of job aids, organization of collaborative learning sessions, etc.), positive perceptions of the package of interventions (i.e. that their ability to perform their job improved since the introduction of the collaborative learning sessions), and the general request of study participants to extend the intervention to other HWs and health facilities. Furthermore, quantitative and qualitative findings converge in suggesting that the package of interventions was effective in helping foster an enabling work environment that improved the motivation and performance of HWs, as evidenced by general positive perceptions of the components of the intervention package – including supportive supervision and collaborative learning – and related improvements reported as far as communication, mentorship and receipt of constructive feedback between managers and HW; and motivation, confidence, and collaboration of HWs within the work environment. Similarly, findings from mystery client interviews and the responses of the majority of respondents to the HW survey suggest that the package of interventions positively improved the attitudes and knowledge of HWs towards providing SRH services to AY related to contraceptive counseling, STIs and IPV.

The findings of this study contribute to the growing area of research concerning the feasibility, acceptability, and effectiveness of interventions to improve the knowledge, skills, attitudes, and performance of HWs in providing SRH services to AY [3]. Our findings align with existing literature which indicates that comprehensive interventions and approaches are more effective than standalone approaches in improving HW performance, including the importance of supportive supervision and collaborative learning as key components in improving health service delivery.^1,2^ In relation to judgmental attitudes among HWs, our findings echo those of others which indicate a need for continuous training and peer/supervisor support as well as incentives and disincentives to address these issues effectively [3].

Our findings have broad implications for future research efforts aimed at further strengthening the evidence base on what works in terms of improving and maintaining the performance of HWs. For instance, rigorous mixed methods hybrid effectiveness and implementation research studies are needed that use study designs which allow for comparisons to be made across participating and non-participating (control) health facilities, as well as advanced statistical analyses which allow for better understanding of which components of an intervention are most effective. Additional qualitative data can also contribute to better understanding of how to address certain factors that influence HW performance including difficulties in communication with AY clients and their parents/guardians, negative attitudes about adolescent SRH and behaviors, and lack of professional motivation linking with a reluctance to change working methods and clinic arrangements [22].

Strengths of the study include how components of the intervention are aligned with evidence-based recommendations concerning HW behavior change for SRH service provision. Specifically, the intervention of the present study has a focus on both HW and AY client-side aspects, goes beyond training to address the physical and social environment, and supports rather than blames HWs [3]. Furthermore, this study was developed within implementation in a real world context. However, with respect to limitations, the study was only conducted in two provinces in the DRC and with a limited sample size of participants, and thus, the findings may not be transferable to other settings in the DRC and other countries as well. Additionally, the lack of baseline measures and a control group constitute additional limitations of this study. Finally, a key contributor to HW performance is the availability of medicines and supplies, medical equipment, and facilities. A key determinant of the use of services is the ability to pay for them or the availability of subsidized/free services. As mentioned previously, these aspects were outside the scope of this intervention.

Future efforts and rounds of implementation of the package of interventions should build on this experience to include a package of approaches including ongoing training and support systems such as collaborative learning that may contribute to addressing technical questions that arise in the course of work, and to reinforcing positive attitudes and practices. The need to go beyond an individual focus to address persistent social norms that shape and hinder HW’s attitudes [23] and practices is vital to ensuring that SRH services respond to the needs and preferences of AY (e.g. to overcome biases that prompt HWs to avoid or provide incomplete SRH counseling to adolescents about contraception and family planning) [22]. Future implementation research should consider collecting longitudinal data from participants to mitigate potential bias, incorporating measures to assess the sustainability and long-term impact of the package of interventions, and include a comparison group (i.e. control health facilities that do not benefit from the intervention activities) to compare measures of feasibility, acceptability and effectiveness. Furthermore, additional research among a more diverse range of participants, particularly adolescent clients, to gauge the perceptions of intervention activities and SRH services by AY would help contribute to understanding of their needs and preferences.

This implementation research indicates that the package of interventions is promising and could be scaled nationally and expanded to other regions through various adaptation strategies. The different components of the intervention – including job descriptions, trainings and periodic refresher trainings, desk reference tools, and collaborative learning sessions – can be replicated in any healthcare setting. However, adaptation strategies for different contexts must consider the reality in many settings – including limited resources and other competing priorities for resources, including staff time. Adaptation strategies, if sufficient resources are available, can include tailoring the package of intervention to other settings to suit the local context. Furthermore, with continued investments, a larger, sustainable workforce that educates and trains subsequent generations of health managers and workers, and which moves beyond one-off training can be developed. We propose several concrete recommendations that address the potential for integration and sustainability of the package of interventions within the health system to avoid dependence on external funding. These are framed around leveraging and embedding in existing structures, fostering local ownership, and ensuring continuous monitoring, evaluation and adaptation of the intervention components. To enhance the sustainability and institutionalization of the package of interventions within the health system, future efforts should focus on: i) ensuring the package of interventions aligns with and is integrated within national health policies on AY SRH, advocate for it to be included in budget allocations and utilize existing health systems resources, ii) capacity strengthening of local HWs and managers through embedding the collaborative learning and supportive supervision components within the existing HW training and support programs, iii) fostering ownership through adolescent, community and stakeholder engagement which can also contribute to increased service utilization and improved health outcomes, and iv) robust monitoring and evaluation to continuously assess the effectiveness of the package of interventions and refine it as needed to demonstrate its value to policymakers and potential funders.

## Conclusions

Our study contributes to the growing body of evidence supporting multi-component interventions to improve HW performance in providing quality SRH services to AY. The findings are promising in that they indicate that the package of interventions was overall perceived to be feasible, acceptable and effective by health facility managers, HWs and AY. We anticipate that the findings from our study on improving the competencies and attitudes of individual HWs and on creating a supportive physical and social environment to motivate and enable them to apply their competencies to the best of their abilities may be useful to guide health programs working to improve the performance of individual HWs and teams of HWs and ancillary staff involved in the care of AY in the DRC and other similar settings. Such initiatives should also consider including an implementation research component to test, refine, adapt and scale-up evidence-based approaches with quality and equity to meet the needs and preferences of AY.

## Supplementary Material

Supplemental Material

## Data Availability

The data that support the findings of this study are available from the study authors upon request.
