# Supplementary material for: Feasibility, acceptability, and effectiveness of a package of interventions to improve the performance of health workers in the Democratic Republic of Congo to deliver adolescent and youth sexual and reproductive health services
Source: Glob Health Action. 2025 Aug 26;18(1):2540687. doi: 10.1080/16549716.2025.2540687 (PMC12381978; doi:10.1080/16549716.2025.2540687)
Supplement: Supplemental Material [file ZGHA_A_2540687_SM1340.docx]

**Title:** Feasibility, acceptability, and effectiveness of a package of interventions to improve the performance of health workers in the Democratic Republic of Congo to deliver adolescent and youth sexual and reproductive health services

**Authors:** Eric Mafuta^1*^ (eric.mafuta@unikin.ac.cd), Kasra Zarei^2*^ (kasra.zarei@ki.se), Landry Egbende^3^ (egbendelandry@gmail.com), Benito Kazenza^3^ ([benito.kazenza@unikin.ac.cd](mailto:benito.kazenza@unikin.ac.cd)), Sheri Bastien^4-6^ (sbastien@who.int), Venkatraman Chandra-Mouli^6-7^ ([chandramouli@bluewin.ch](mailto:chandramouli@bluewin.ch))

*Denotes joint first authorship

**Affiliations:**

1. Department of Health Systems Policy and Management, University of Kinshasa School of Public Health, Kinshasa, Democratic Republic of the Congo
2. Department of Global Public Health, Karolinska Institutet, Stockholm, Sweden
3. Department of Nutrition, University of Kinshasa School of Public Health, Kinshasa, Democratic Republic of the Congo
4. Department of Public Health Science, Norwegian University of Life Sciences, Ås, Norway
5. Cumming School of Medicine, Department of Community Health Sciences, University of Calgary, Calgary, Canada
6. Department of Sexual and Reproductive Health and Research, World Health Organization, Geneva, Switzerland
7. Independent learner, communicator, advisor, teacher, and supporter of adolescent advocacy, research, and action, Geneva, Switzerland (formerly)

**Corresponding Author:** Kasra Zarei, email: kasra.zarei@nih.gov; postal address: 6707 Democracy Boulevard, Suite 800 Bethesda, MD 20892-5465

**SUPPLEMENTAL**

**Introduction**

***Study Context***

In sub-Saharan Africa and other areas of the world, adolescents, and particularly adolescent girls, are vulnerable to sexually transmitted infections (STIs) such as HIV, unwanted pregnancies, and other adverse sexual and reproductive health (SRH) outcomes due to individual and systemic risk factors.(1-3) The Democratic Republic of the Congo (DRC) has the fourth highest population in Africa and has a large number of youth, with an estimated 47% of the population under the age of 18, aged 0-14 according to the World Bank.(4) In the DRC, adolescent girls are at a disproportionate risk of poor adolescent SRH outcomes due to risk factors including early sexual debut, unprotected sexual activity, transactional sex, and violence, including sexual violence.(5, 6) Efforts to improve adolescent SRH outcomes are needed to meet the United Nations Sustainable Development Goals (SDGs), particularly “Good Health and Well-Being” (SDG 3) and “Gender Equality”.” (SDG 5). While the DRC is on track and/or maintaining the SDG achievements of reducing new HIV infections – with a decrease in new HIV infections across all ages in recent decades – and its ratio of female-to-male labor force participation, major challenges remain for achieving the other SDGs and indicators. including ensuring inclusive and equitable quality education for all.(7)

**Methodology**

***Training of Research Team Members***

Research team members were trained for the project in three phases. The training of the research teams to collect data from HWs and facility managers began with an online training (two hours per day for four days) in 2020-2021, carried out by the World Health Organization (WHO) manager in charge of the study and the consultant in charge of supporting the research team. During these sessions, the study objectives, methodology, and data collection tools were presented and explained to the members of the research team. The trainings were participatory to allow the various actors involved to quickly familiarize themselves with the study methodology, the data collection tool, and the environment surrounding the study.

The research team then organized the training of investigators in charge of carrying out health visits by mystery clients within the health facilities involved in the project, and the training for mystery clients to collect information to evaluate their perceptions and experiences of the AY SRH health care and services received by HWs. The third phase of the training concerned the exit interviews to assess the satisfaction of AY with the services provided in the health facilities. The training lasted three days in April-June 2021 and took place face-to-face at both sites to bring together all the research team members.

***Initial Validation of Data Collection Tools***

After translating the data collection tools from English into French, the research team pre-tested them in an environment like that of the intervention to assess the validity and accuracy of the questions, the administration time of the different tools, the presentation of the questions to respondents, translation fidelity and questions administration issues as well as interviewers' and respondents' perception of the questions. The pre-test took place in an environment like that of the intervention, while using role-playing simulations in situations involving AY exit interviews or mystery clients. Field difficulties were shared by the investigators with the study coordination team. These difficulties related to the availability of AY for exit interviews and of HWs. Recommendations resulting from this preliminary validation included the need to: 1) clarify certain concepts such as the collaborative learning model with investigators and other members of the research team during the training or retraining of investigators, given that not everyone had access to the document produced by the National Adolescent Health Program on the theme, 2) reduce interview time for some modules, particularly the one for AY and the HW questionnaire, 3) identify beforehand, for the health facilities that will be selected, the consultation days dedicated to AY in order to have the greatest number of AY.

**REFERENCES**

1. Melesse DY, Mutua MK, Choudhury A, Wado YD, Faye CM, Neal S, et al. Adolescent sexual and reproductive health in sub-Saharan Africa: who is left behind? BMJ Glob Health. 2020;5(1):e002231. Epub 20200126. doi: 10.1136/bmjgh-2019-002231. PubMed PMID: 32133182; PubMed Central PMCID: PMC7042602.

2. Janighorban M, Boroumandfar Z, Pourkazemi R, Mostafavi F. Barriers to vulnerable adolescent girls' access to sexual and reproductive health. BMC Public Health. 2022;22(1):2212. Epub 20221129. doi: 10.1186/s12889-022-14687-4. PubMed PMID: 36447192; PubMed Central PMCID: PMC9706928.

3. World Health Organization & UNDP/UNFPA/UNICEF/WHO/World Bank Special Programme of Research, Development and Research Training in Human Reproduction. Sexual health and its linkages to reproductive health: an operational approach. World Health Organization, 2017.

4. The World Bank in the Republic of Congo 2024. Available from: <https://www.worldbank.org/en/country/congo/overview>.

5. Ninsiima LR, Chiumia IK, Ndejjo R. Factors influencing access to and utilisation of youth-friendly sexual and reproductive health services in sub-Saharan Africa: a systematic review. Reprod Health. 2021;18(1):135. Epub 20210627. doi: 10.1186/s12978-021-01183-y. PubMed PMID: 34176511; PubMed Central PMCID: PMC8237506.

6. Decker MR, Latimore AD, Yasutake S, Haviland M, Ahmed S, Blum RW, et al. Gender-based violence against adolescent and young adult women in low- and middle-income countries. J Adolesc Health. 2015;56(2):188-96. doi: 10.1016/j.jadohealth.2014.09.003. PubMed PMID: 25620301.

7. Sachs JD, Lafortune G, Fuller G, Drumm E. Implementing the SDG Stimulus. Sustainable Development Report 2023. Paris: 2023.

**TABLES**

**Supplemental Tables**

**Supplemental Table 1.** Summary of sample recruited for study

| **Data collection technique** | **Kinshasa** | **Mbuji-Mayi/Eastern Kasai** | **Total** |
| --- | --- | --- | --- |
| Individual interview of health facility managers | 10 | 15 | 25 |
| Focus groups of health workers | 2 | 2 | 4 |
| Rating of mystery clients | 6 | 10 | 16 |
| Exit interviews with teenage clients | 8 | 10 | 18 |
| Structured interviews with health workers | 24 | 30 | 54 |

**Supplemental Table 2.** Characteristics of study participants by research project activity in 2020

| Activities | Total | Sex | | Sites | | Age | | | Education Level | |
| --- | --- | --- | --- | --- | --- | --- | --- | --- | --- | --- |
|  |  | Male | Female | Mbuji-Mayi | Kinshasa | ≤ 19 | 20-25 | >25 | Secondary school | >Secondary School |
| Interviews with managers of health facilities | 25 | 13 | 12 | 15 | 10 | 0 | 0 | 25 | 9 | 16 |
| Focus Group discussion | 29 | 17 | 12 | 16 | 13 | 0 | 4 | 25 | 2 | 27 |
| Structured interviews | 54 | 20 | 34 | 30 | 24 | 0 | 11 | 43 | 11 | 43 |
| Mystery clients | 16 | 0 | 16 | 10 | 6 | --- | --- | --- | --- | --- |
| Exit Interviews | 18 | 0 | 18 | 10 | 8 | --- | --- | --- | --- | --- |

*To preserve anonymity, the number of mystery clients and exit interviews based on category of age and education level are not reported due to small cell counts.
